# Antimicrobial resistance and genomic characterization of *Staphylococcus aureus* in ready-to-eat foods from Mangaung Metro Municipality

**DOI:** 10.3389/fmicb.2025.1669035

**Published:** 2025-12-05

**Authors:** Pontso Letuka, Sebolelo J. Nkhebenyane, Tsepo Ramatla, Tywabi-Ngeva Zikhona, Kgaugelo E. Lekota, Ntelekwane G. Khasapane

**Affiliations:** 1 Department of Life Sciences, Centre for Applied Food Safety and Biotechnology, Central University of Technology, Bloemfontein, South Africa; 2 Department of Chemistry, Nelson Mandela University, Port Elizabeth, South Africa; 3 Unit for Environmental Sciences and Management, North-West University, Potchefstroom, South Africa

**Keywords:** *Staphylococcus aureus*, WGS, antibiotic resistance, ready-to-eat food, virulence factors

## Abstract

Human staphylococcal food poisoning is caused by a range of heat-stable staphylococcal enterotoxins that are released into food by *Staphylococcus aureus*. Additionally, antimicrobial resistance has steadily grown into a significant global issue that endangers food safety and human health. Therefore, this study aimed to determine the frequency of *S. aureus* in foods sold on the streets of Mangaung Metropolitan Municipality, South Africa, and thoroughly examine the isolated strains’ genetic traits, virulence, and antibiotic resistance profiles. Out of 168 samples, which included salad, pap (maize meal), chicken, and pork, all (100%) samples showed the occurrence of *Staphylococcus* species. Furthermore, 29.7% of isolates were subsequently identified by MALDI-TOF MS as *S. aureus*. Moreover, antimicrobial susceptibility testing of 50 *S. aureus* isolates showed that 42% were resistant to penicillin, followed by cefoxitin at 46% and ciprofloxacin at 44%. The multidrug-resistant (MDR) profile revealed that 52% of the isolates were resistant to three or more classes of antibiotics. Additionally, four sequenced isolates were identified by in silico MLST as having sequence types (STs) 243, although strain SVF3 contained a unique ST designated as * ff2b. All four isolates were identified as belonging to Staphylococcal Protein A type (*spa-type*) t21 by whole genome sequencing. All sequenced isolates exhibited a total of 9 antibiotic resistance genes and 63 virulence genes. The current study showed the importance of monitoring for high virulence potential and antimicrobial resistance of *S. aureus* in retail food and increasing awareness of potential risk for such strains; furthermore, infection control measures, antimicrobial stewardship, and periodic One Health epidemiological surveillance studies are needed to monitor and contain the threat of increasing antibiotic resistance in Africa.

## 1 Introduction

Street food vending has recently expanded, resulting from many surrounding socio-economic factors. The need to provide employment, boost household income, and provide affordable, convenient varieties of meals has contributed to a boom in the street food industry (Salamandane et al., 2023). However, street food vending in developing countries is mainly unregulated and managed informally (Desye et al., 2023). As a result, the street food vending trade involves unhygienic and unsanitary practices, lack of sanitary facilities, and use of poor-quality equipment (Akhter and Cameron, 2023). This has had a domino effect on the safety of the foods produced, with many concerns being raised about their health risks to consumers. The World Health Organization (2022) estimated that one in ten people worldwide develops an illness from consuming contaminated food annually. In South Africa and other parts of the world such as Indonesia, *S. aureus* is among the frequently reported foodborne pathogens (Mazizi et al., 2017; Thaha et al., 2024; Thwala et al., 2022).

*Staphylococcus aureus* is responsible for causing foodborne illnesses on a global scale. Its ability to produce toxins in contaminated food can lead to severe food intoxication, affecting individuals and communities worldwide (Fetsch and Johler, 2018). *S. aureus* and other *Staphylococcus* spp. exist as part of normal human flora and are commonly found in the nose and the perineum. Therefore, when food handlers fail to practice good hygiene, such as handwashing with running water and soap, they can contaminate the food they touch. Previous studies have indicated that *S. aureus* can survive and proliferate on food and contact surfaces, creating perfect cross-contamination opportunities (Castro et al., 2018; Bai et al., 2023).

In the food industry, control measures of *S. aureus* and other pathogens primarily focus on hygiene strategies. However, because *S. aureus* is a biofilm-forming organism, and hygiene may be poor in street food vending, it can be challenging to eliminate it (Saad et al., 2023; Letuka et al., 2021). Moreover, its virulence factors and antibiotic resistance make it a particularly worrisome pathogen in food safety (Fernandes et al., 2022). To obtain the ecological and epidemiological characteristics of *S. aureus* implicated in food poisoning, numerous molecular techniques such as coagulase typing, staphylococcal protein A gene (*spa*) typing, and pulsed-field gel electrophoresis (PFGE) have commonly been employed (Roussel et al., 2015). While these techniques provide valuable insights into the spread, origin, and genetic analysis of *S. aureus*, they often lack the resolution necessary to detect genetic differences between strains (Khasapane et al., 2024). Moreover, whole genome sequencing has emerged as the preferred method due to its superior capability to enhance understanding of phylogenomics and intraspecies variations (Khasapane et al., 2024; Lekota et al., 2024).

Given the global context and the variety of *S. aureus* strains present in South Africa, including virulent and antibiotic-resistant lineages, there is a significant need for large-scale genomic analyses of local *S. aureus* isolates (Ndhlovu et al., 2024). Such studies would enhance our understanding of the regional pan-genome, revealing unique genetic elements and potential public health concerns. Ongoing genomic surveillance is crucial for tracking the emergence and spread of virulent or resistant strains, which can inform infection control strategies and treatment guidelines (Piper et al., 2024). While specific pangenomic studies of *S. aureus* in South Africa are limited, current research highlights this pathogen’s genetic diversity and adaptability in the region. Comprehensive local studies are essential to fully understand the pangenome landscape and its implications for public health.

*Staphylococcus aureus* displays considerable genetic variation, which enhances its capacity to inhabit a wide range of hosts and environments (Wang et al., 2017). This variation is evident in its pan-genome and contains many genes related to antibiotic resistance (Moller et al., 2022). Street-vended foods are frequently prepared and sold in conditions that may not adhere to rigorous hygiene standards. This lack of proper sanitation can result in contamination by pathogenic bacteria, such as *S. aureus*, which is known to cause foodborne illnesses (Amare et al., 2019). Very few studies have been done to assess the microbial profile of street-vended foods in the Mangaung Metropolitan Municipality, and none have gone as far as to characterize and define the genetic variations of food microorganisms found in ready-to-eat foods in this area. Therefore, the purpose of this study was to effectively assess the prevalence of *S. aureus* in street-vended foods, along with a comprehensive analysis of the genomic characteristics and antimicrobial resistance of the isolated strains.

## 2 Materials and methods

### 2.1 Study site and collection of samples

**Figure 1:**
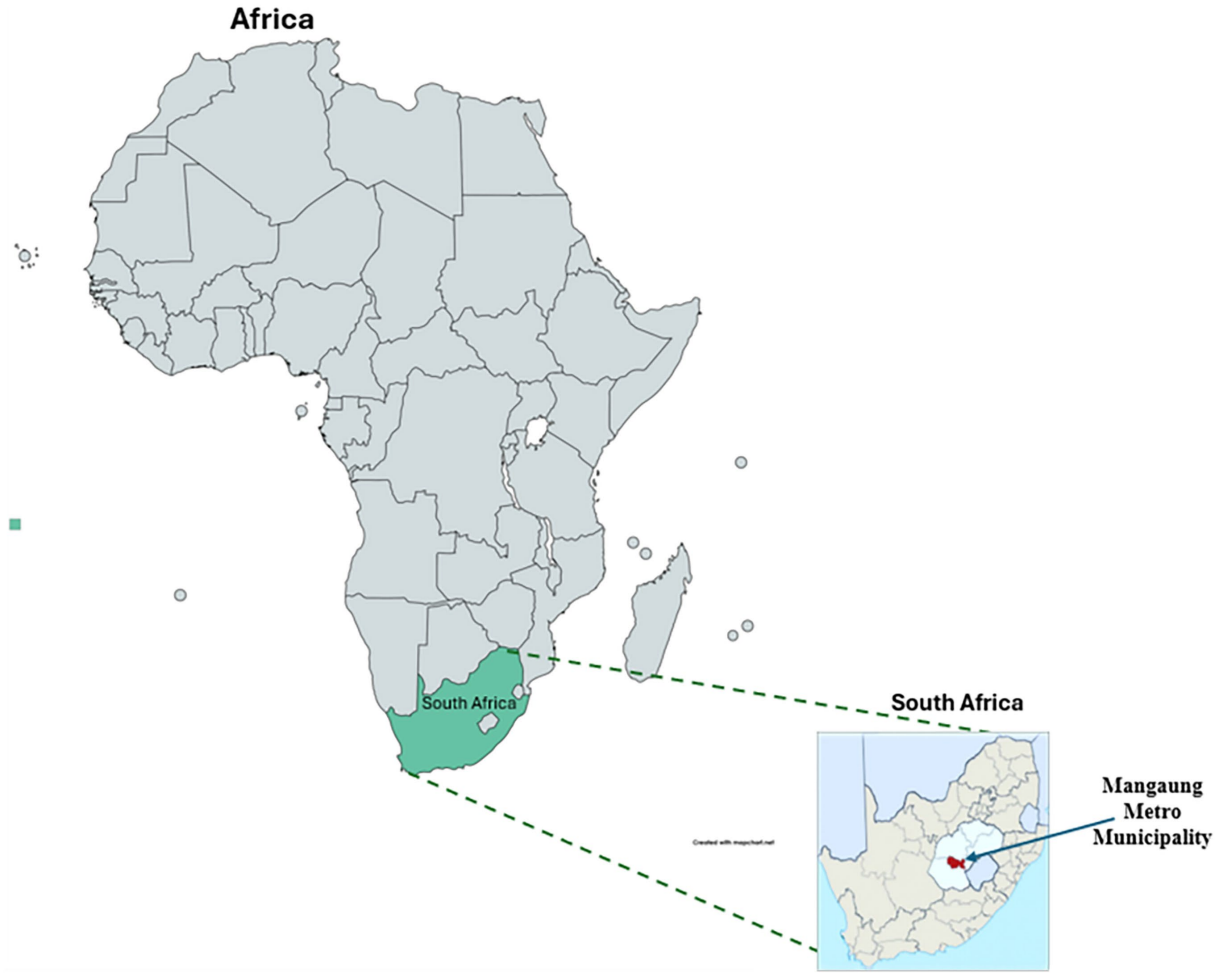
South African map showing Free State province, red color indicates [Mangaung Metro Municipality] where samples were collected.

The current study was conducted in Mangaung Metro Municipality, focusing on its seven main areas: Bloemfontein, Botshabelo, Thaba Nchu, Wepener, Dewertsdop, Soutpan, and Van Stadensrus (Figure 1). Mangaung Metro Municipality is a Category A municipality nestled in the central interior of South Africa in the Free State province (29.1303^o^S, 26.2358°E).

A total of 168 samples were collected from 4 different food types typically found in this study area: maize meal/pap (42), chicken (42), pork (42), and salad (42) between March and April 2023. The number of samples was determined by the logistical feasibility, resources available, and the need to obtain a representative sample across the different food types, vendors and location. Using this sample size allowed for a balance in representation without compromising on issues of laboratory and analytical capabilities. The samples were aseptically collected using sterile bags and immediately transported to the Centre for Applied Food Sustainability and Biotechnology, Bloemfontein, for analysis in cool temperatures of 4 °C.

### 2.2 Bacteriological examination

Upon arrival, samples (10 g) were weighed and homogenized in 90 mL buffered peptone water (Thermo Fischer Scientific). To determine *S. aureus*, counting and isolation were done following International Organization for Standardization (ISO) 6888-1: 2021 (ISO, 2021). Tenfold serial dilutions were conducted using buffered peptone water, and 0.1 mL aliquots were cultured on Baird Parker agar supplemented with egg yolk tellurite emulsion (Thermo Fischer Scientific, South Africa). Incubation was done at 35 °C for 24–48 h. One to three well isolated colonies that were morphologically typical of *S. aureus* (black, shiny colonies with clear zones) were purified on nutrient agar (Merck, Wadeville, South Africa) and then incubated at 37 °C for 24 h (Khasapane et al., 2024). After phenotypic identification, the isolates were confirmed as putatively pathogenic *S. aureus* isolates and were subjected to Gram staining and coagulase activity (Alksne et al., 2020). All the purified isolates were stored in Tryptic Soy Broth supplemented with 15% glycerol (Thermo Fischer Scientific, South Africa) at −80 °C for further analysis (Fernandes et al., 2022).

### 2.3 Species identification by MALDI-TOF MS

Utilizing the Biotyper 3.1 tool (Bruker, Johannesburg 2191, South Africa), staphylococcal species or genera were identified. The Autoflex Speed apparatus (Bruker Daltonics, Billerica, MA) was calibrated using the *Escherichia coli* DH5α Bacterial Test Standard (BTS). According to Cameron et al. (2018), every bacterial isolate was also identified. In short, sterile 1.0 μL disposable plastic inoculating loops were used to transfer the pure colony to individual 1.5 mL microcentrifuge Safe-Lock Tubes (Sigma-Aldrich, Brondbyvester, Denmark) that contained 300 μL of ultra-HPLC grade water (Merck, Hellerup, Denmark). After a quick vortex to produce a uniform suspension, 900 μL of 100% ethanol (Merck, South Africa) was added, and the tubes were vortexed for 15 s. After centrifuging the tubes for 3 min at 12,200×*g* at room temperature (RT), the supernatants were drained out and disposed of, and the tubes were centrifuged once more for 3 min at RT. A micropipette was used to aspirate the leftover ethanol/water properly. After allowing the cell pellets to air dry for 3 min, a suitable volume of 70% formic acid (15–50 μL) was added. By visually sizing the pellet, the ideal formic acid volume was identified. Each sample was thoroughly mixed with an equal volume of 100% acetonitrile after up to 3 min. At room temperature, samples were centrifuged at 12,200×*g* for 3 min. After carefully placing eight 1 μL volumes of supernatant on a ground steel target plate and letting it air dry, 1.0 μL of cyano-4-hydroxycinnamic acid (HCCA) matrix (Bruker Daltonics) diluted in 50% acetonitrile with 2.5% trifluoroacetic acid (Sigma-Aldrich) was applied to each spot. Every isolate’s analysis was performed twice. If isolates were not resolved after two rounds of MALDI-TOF MS analysis, they were deemed unidentified. A cut-off score of ≥ 1.7 was employed as a threshold for detecting bacteria to ensure our analysis’s credibility.

### 2.4 Antimicrobial susceptibility testing

For phenotypic susceptibility testing of the isolates, disk diffusion was performed with seven antibiotics on a 90 mm plate (Mekhloufi et al., 2021). Using a sterile cotton swab, 100 μL aliquots from the overnight cultures were spread-plated on Mueller Hinton agar (MH), and the plates were incubated at 37 °C for 24 h. Antibiotic discs (ThermoFisher, South Africa) comprising tetracycline a member of the class of tetracyclines (TE, 30 μg), penicillin (P, 10 μg) and cefoxitin (FOX, 30 μg) belonging to the class of *β*-Lactam, the macrolide erythromycin (E, 15 μg), gentamicin (GEN, 10 μg) of an aminoglycoside class and ciprofloxacin (CIP, 10 μg) a fluoroquinolone class were utilised in this investigation (Islam et al., 2025). The Clinical Laboratory Standards Institute (CLSI) standards (Clinical and Laboratory Standards Institute, 2024), which are classified as intermediate (I), susceptible (S), and resistant (R), were used. Furthermore, strain *Staphylococcus aureus* ATCC 25923 was used as a positive control (CLSI, 2024). Isolates were defined as multidrug-resistant if they exhibited resistance to at least three or more different classes of antibiotics (Khasapane et al., 2024; de Wet et al., 2025). The selection of these antibiotics was predicated on their extensive availability, market presence, and common prescription for bacterial infections, as corroborated by existing literature.

### 2.5 MAR index calculation

The MAR index was calculated and interpreted according to Krumperman (1983) using the formula: *a*/*b*, where ‘*a*’ represents the number of antibiotics to which an isolate was resistant, and *‘b*’ represents the total number of antibiotics tested.

### 2.6 Statistical analysis

The obtained results were statistically evaluated by application of Analysis of Variance (ANOVA) test according to Shaltout et al. (2017).

### 2.7 Molecular characterization

Out of 50 isolates confirmed as *S. aureus* using MALDI-TOF, only four isolates were selected for whole genome sequencing. The selection of these four isolates from each sample type was based on their phenotypic antimicrobial resistance; these included whether they were multidrug resistant and what was the index of their multiple antibiotic resistance. Following the manufacturer’s instructions, the Quick-DNA Microbe Mini-prep DNA kit was used to extract genomic DNA of *S. aureus* (ZYMO Research, USA). The concentration and purity of the extracted DNA were determined using a NanoDrop spectrophotometer (Thermo Scientific, Wilmington, DE, USA). The DNA was sent to an outsourced company for genome sequencing after that using the PacBio sequel II platform at Inqaba Biotechnical Industries (Pty) Ltd. (525 Justice Mahomed St, Muckleneuk, Pretoria, 0002).

### 2.8 Bioinformatics analysis

The quality of sequenced reads generated by the PacBio platform was evaluated using FastQC software version 0.10.1 (Chen et al., 2018). Paired-end trimmed reads were *de novo* assembled using Flye version 2.3.3 (Kolmogorov et al., 2019). CheckM v 1 (Parks et al., 2015) was utilized to identify potential contaminants in individual assembled genomes. Quast version 2.3 (Gurevich et al., 2013) was employed to evaluate the assembled genomes. Subsequently, the assembled contigs were annotated using the NCBI Prokaryotic Genome Automatic Annotation Pipeline (PGAAP) (Tatusova et al., 2016).

The taxonomic classification of bacterial strains was conducted in silico using multilocus sequence typing (MLST), as Carattoli et al. (2014) outlined, and the Pasteur database implemented in PathogenWatch.^1^ GTDBtk v1.7.0 (Chaumeil et al., 2020) was employed to identify *Staphylococcus* species, following the methodology presented by Chaumeil et al. (2020). To determine the taxonomic positions of the *Staphylococcus* strains, DNA–DNA hybridization values were calculated using formula d4, and a phylogenomic tree based on whole genome sequencing (WGS) was reconstructed using the Type Strain Genome Server (TYGS), as described by Meier-Kolthoff and Göker (2019). This method calculates the DNA similarity between aligned fragments of a query genome and a Type Strain database, using a similarity cutoff of over 70% for identifying the same species. Average nucleotide identity (ANI) was used to determine the genetic relatedness of the strains using IPGA (Varghese et al., 2015). *Spa types* were identified using the Centre for Genomic Epidemiology online tool.^2^

The pangenome analysis of the dispensable genomes was conducted using Roary v3.6.8 (Page et al., 2015) and Anvio-7.1 (Eren et al., 2015). Eighty-four *S. aureus* genomes from South Africa were downloaded from BV-BRC^3^ and compared with the four sequenced isolates in this study (Supplementary Table S1). The genomes were annotated using Prokka version 1.14 (Seemann, 2014) and Prodigal (Hyatt et al., 2010). Pairwise BLASTp and the Markov Cluster Algorithm (MCL) were employed for similarity searches between the coding domain sequences (CDSs) of the assembled genomes, following the pipeline outlined by Magome et al. (2024). Pangenome clusters were defined as follows: Core genes are those present in all isolates. Soft core genes are present in at least 95% of isolates. Shell genes are found in between 15 and 95% of isolates, while cloud genes exist in less than 15% of isolates. The assignment of clusters of orthologous groups (COG) was accomplished using DeepNOG version 1.2.3 (Feldbauer et al., 2020).

The ABRicate pipeline (assessed on 25 July 2024) and AMRFinderplus (Feldgarden et al., 2022) were utilized to identify antibiotic resistance and virulence genes in the genome of *S. aureus*. Antimicrobial resistance determinants were detected in the assembled genome using the Comprehensive Antibiotic Resistance Database (CARD) database (Hackenberger et al., 2025), applying minimum identity and coverage thresholds of 75% (−minid 75) and 50% (−mincov 50), respectively. Moreover, the Comprehensive Antibiotic Resistance Database (CARD) was employed to identify antibiotic resistance genes. ABRicate was also used to analyze efflux pump coding genes and virulence factors in the sequenced genome, utilizing the Virulence Factor Database (Liu et al., 2022) with minimum identity and coverage thresholds of 70% (−minid 70) and 50% (−mincov 50), respectively. Additionally, plasmid replicons were identified in the sequenced genomes by ABRicate using the Plasmid Finder database (see text footnote 2). The profiles were visualised using circos plots (Krzywinski et al., 2009).

## 3 Results

### 3.1 Identification of *Staphylococcus* species isolates

The findings of this study revealed that out of 168 samples, which included pap, chicken, pork, and salad, all samples showed the presence and enumeration of *Staphylococcus* species when examined morphologically (Table 1). Thereafter, one colony was picked from each of the samples for further identification by MALDI-TOF MS. The results showed that 50/168 (29.7%) isolates were then identified as *S. aureus* by MALDI-TOF MS (Table 2). In addition, the results showed that the highest number of isolates was obtained from pork with 34 (39.5%) isolates, followed by chicken with 26 (30.2%) and the lowest number was obtained from pap with 14 (16.2%) isolates and salad with 12 (13.9%) isolates, both using the two identification methods with *p* values of 0.010, 0.019, 0.012 and 0.013 from food samples, however, regional analysis showed that Thaba Nchu, Wepener, Van Stadensrus and Bloemfontein had the highest number of cultured colonies with *p* values of 0.0.031, 0.012, 0.027 and 0.150 as compared to Botshabelo (*p* < 0.055), Dewertsdorp (*p* < 0.022) and Soutpan (*p* < 0.058).

**Table 1:** Presumptive microbial counts of *Staphylococcus aureus* obtained from different food samples.

Sampling areas	Food samples				Statistical analysis	
	Pap	Chicken	Pork	Moroho/Salad	Mean	*p* value
Bloemfontein	7.2 × 10^5^ CFU/g	1.2 × 10^5^ CFU/g	1.7 × 10^4^ CFU/g	1.2 × 10^4^ CFU/g	282.5	0.150
Botshabelo	1.8 × 10^3^ CFU/g	1.2 × 10^3^ CFU/g	3.7 × 10^4^ CFU/g	2.1 × 10^5^ CFU/g	220	0.055^*^
Thaba Nchu	7.5 × 10^4^ CFU/g	1.3 × 10^5^ CFU/g	7.8 × 10^5^ CFU/g	4.9 × 10^4^ CFU/g	537.5	0.031^*^
Wepener	2.0 × 10^3^ CFU/g	3. × 10^3^ CFU/g	3.4 × 10^3^ CFU/g	4.6 × 10^3^ CFU/g	325	0.012^*^
Dewertsdorp	1.5 × 10^4^ CFU/g	1.7 × 10^4^ CFU/g	2.4 × 10^4^ CFU/g	1.4 × 10^4^ CFU/g	175	0.022^*^
Soutpan	2.3 × 10^3^ CFU/g	1.9 × 10^3^ CFU/g	1.4 × 10^2^ CFU/g	4.6 × 10^4^ CFU/g	255	0.058^*^
Van Stadensrus	6.1 × 10^2^ CFU/g	3.9 × 10^2^ CFU/g	4.7 × 10^2^ CFU/g	1.4 × 10^3^ CFU/g	402.5	0.027^*^
Mean	405.71	202.85	358.57	288.57	–	–
*p* value	0.012^*^	0.019	0.010^*^	0.013^*^	–	–

^*^Statistically significant.

**Table 2:** The number of samples collected per Mangaung Metro Municipality and the positive *Staphylococcus* spp. isolates.

Samples	Number of samples	MALDI-TOF MS
Pap	42	11
Chicken	42	16
Pork	42	18
Salad	42	5
Total	168	50

### 3.2 Antimicrobial resistance profiles of the *Staphylococcus aureus*

**Figure 2:**
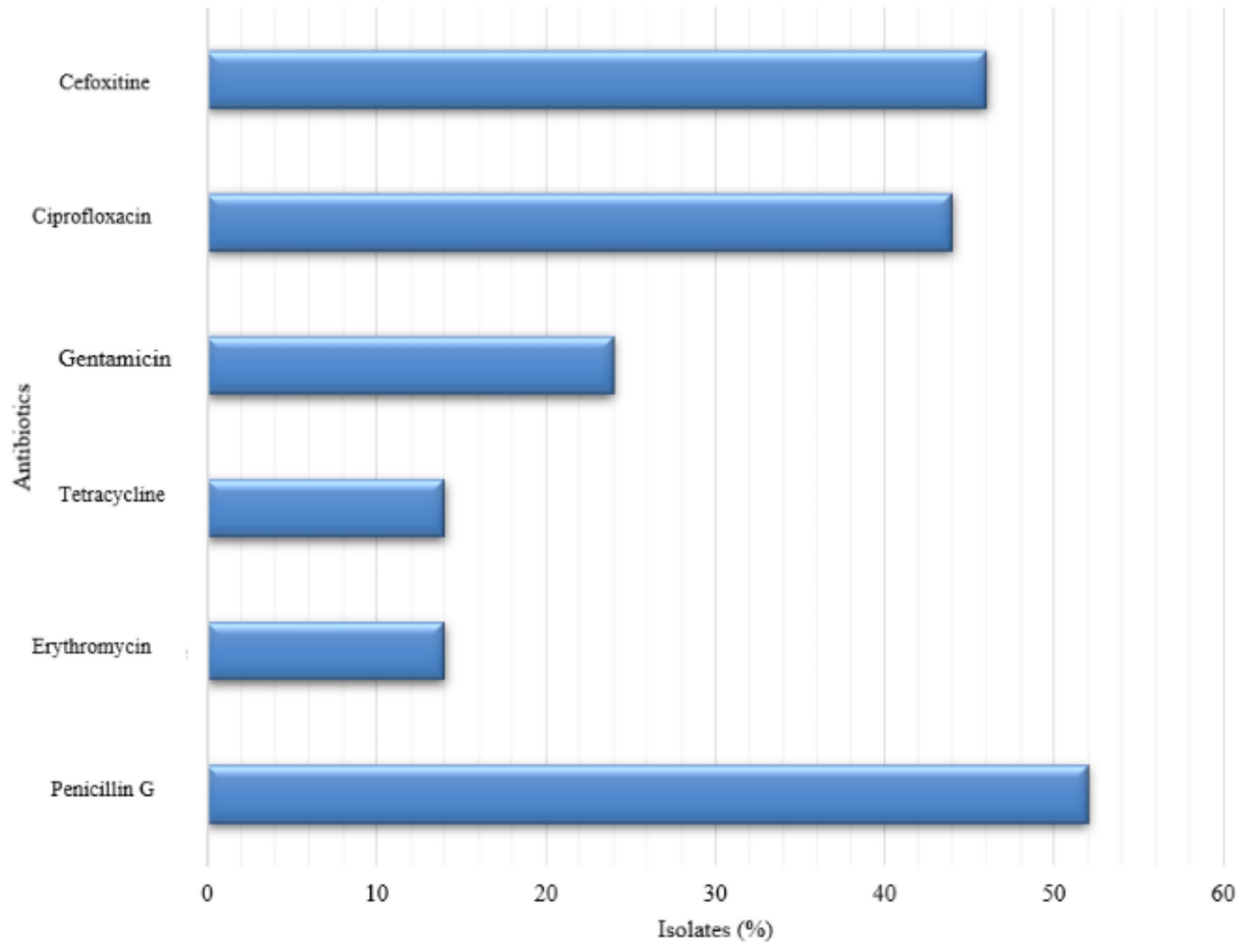
Antibiotic resistance patterns of *S. aureus* isolated from ready-to-eat food in Mangaung Metro Municipality.

Furthermore, our phenotypic antimicrobial susceptibility testing showed that 26/50 (52%) of the isolates were resistant to penicillin, followed by cefoxitin with 23/50 (46%) and ciprofloxacin with 22/50 (44%). In contrast, lower resistance rates could be noticed for gentamicin (12/50, 24%) and for both erythromycin and tetracycline at (7/50, 14%) (Figure 2).

The multidrug-resistant results revealed that 21/50 (52%) of the isolates were resistant to three or more classes of antibiotics. Table 3 shows that 6/21 (12%) of the isolates were resistant to three antibiotics, including P, CIP and FOX. Moreover, one isolate (2%) was resistant to 6 antibiotics, whereas seven isolates were resistant to at least four antibiotics. In total, 23 isolates showed phenotypic MDR with at least 3 different antimicrobial classes. Of these, 11 (52.3%), 5 (23.8%), 4 (19%) and 3 (14.2%) were sourced from poultry, pork, pap and salad, respectively.

**Table 3:** Antibiotic resistance patterns of *S. aureus* isolated from Mangaung Metro Municipality.

	Sample ID	*β*-lactams		Macrolide	Tetracycline	Aminoglycoside	Fluoroquinolones
		Penicillin	Cefoxitin	Erythromycin	Tetracycline	Gentamycin	Ciprofloxacin
Pap	P1	R	R	R	R	S	R
	P2	R	S	S	S	S	R
	P3	S	S	R	S	R	R
	P4	R	S	S	S	R	R
	P5	R	S	S	S	R	R
	P6	R	R	S	R	R	-
	P7	R	S	R	S	S	R
	P8	R	R	S	S	S	S
	P9	S	R	S	R	S	R
Chicken	C10	S	S	R	S	S	R
	C11	R	S	S	S	R	R
	C12	R	R	S	S	S	R
	C13	R	S	S	S	S	R
	C14	R	S	S	S	R	R
	C15	R	S	S	R	R	R
	C16	R	R	S	S	R	R
	C17	R	R	S	S	S	S
	C18	R	R	S	S	S	R
	C19	R	R	S	R	S	R
	C20	R	R	S	S	S	S
	C21	R	S	S	R	S	R
	C22	R	S	S	S	S	R
	C23	R	S	S	S	R	S
	C24	R	S	S	S	S	R
	C25	R	S	S	R	S	R
	C26	R	S	S	S	S	R
	C27	R	S	S	S	S	S
	C28	R	R	R	S	S	S
	C29	R	S	S	S	R	R
	C30	R	R	S	S	R	R
	C31	R	S	S	S	R	R
	C32	R	S	S	S	S	S
Pork	PK33	R	S	S	S	S	R
	PK34	R	S	S	S	R	R
	PK35	R	R	S	R	S	S
	PK36	R	S	S	S	R	R
	PK37	R	R	R	R	S	R
	PK38	R	R	S	S	R	R
	PK39	R	R	S	S	S	R
	PK40	R	R	S	S	R	R
	PK41	R	R	S	S	S	R
Salad	S42	S	R	R	R	S	S
	S43	R	R	S	S	S	R
	S44	S	R	S	S	R	R
	S45	S	R	S	S	S	S
	S46	R	S	S	S	R	R
	S47	R	R	R	S	R	R
	S48	S	R	R	S	S	R
	S49	R	R	S	S	S	S
	S50	R	R	S	S	S	R

### 3.3 MAR index

The results of MAR index indicated a high index on isolates P1, PK37 and S47 with 0.83, while isolate C15 showed an index of 0.66 (Supplementary Table S1). Therefore, the results indicate a high risk of antibiotic resistance.

### 3.4 Genomic features and *in silico* identification

In this study, we sequenced the genomes of four isolates (P1, C15, PK37, and S47) based on their MDR patterns from Table 3, and the characteristics of their genomes are shown in Table 4. The sequenced strains’ genome size ranged from 2.6 Mb to 2.8 Mb, with a GC content of 32.9%. The application of TYGS categorizes these isolates of *S. aureus* alongside *S. aureus* DSM 20231 (Supplementary Figure S1). The number of coding sequences ranged from 2,626,607 to 2,855,328 sequences.

**Table 4:** Genomic traits of four sequenced *S. aureus* strains isolated from food.

Strain name	BioSample accession no.	GenBank accession no.	Completeness	No. of raw reads	No. of contigs	Coverage depth	N_50_ value (bp)	Genome length (bp)	Total no. of genes
SVF1 (P1)	SAMN45157884	SUB14878302	97,37	70,158	5	140x	2,489,803	2,855,949	2,673
SVF2 (C15)	SAMN45158357	SUB14908207	98,72	91,766	3	140x	2,237,147	2,833,403	2,668
SVF3 (PK37)	SAMN45158359	SUB14908216	91,28	130,620	4	140x	2,196,342	2,627,141	2,445
SVF4 (S47)	SAMN45158360	SUB14908224	99,51	115,379	3	140x	2,227,546	2,836,470	2,650

**Figure 3:**
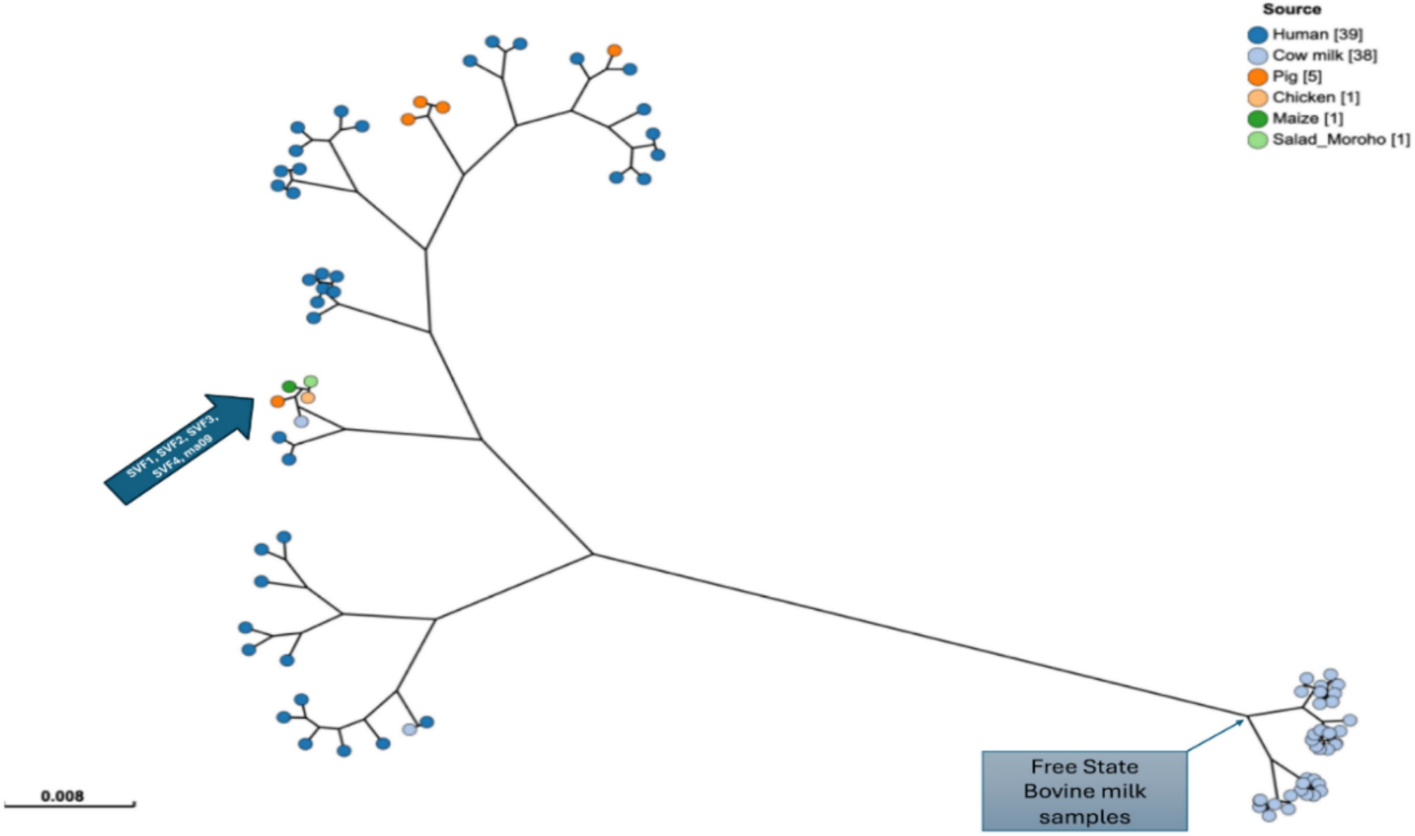
Multi locus sequence typing indicating the clustering of the 85 *S. aureus* with host source in South Africa. The color of the strains is grouped according to sources. The phylogenetic tree was computed using the maximum likelihood of whole genome core single nucleotide polymorphisms.

**Figure 4:**
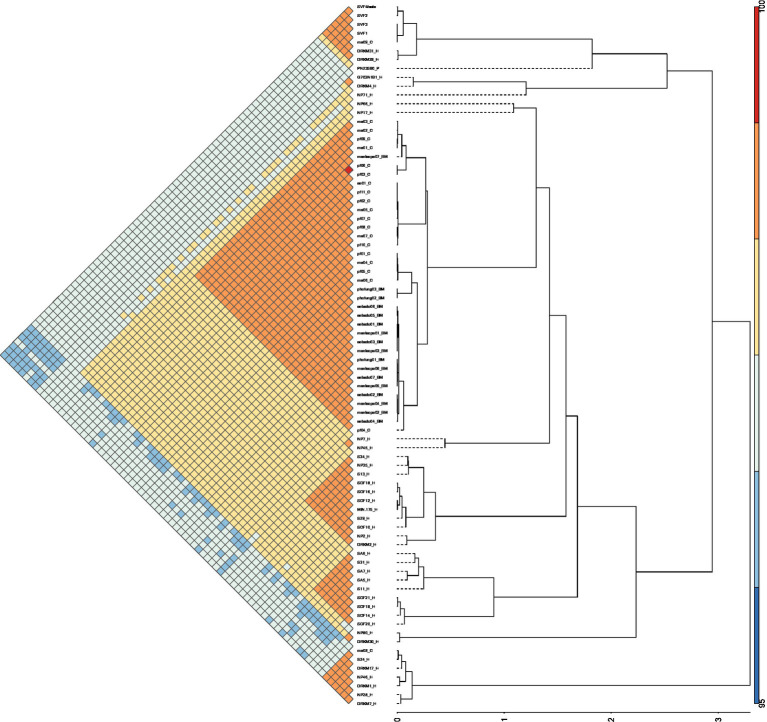
Average nucleotide identity of the 85 *S. aureus*, including the four sequenced genomes in this study. The strain names are designated as follows: _H represents human, _C denotes cow, and BM indicates bovine mastitis-associated isolates. The sequenced genome strains are designated as SVF1, SVF2, SVF3, and SVF4, which cluster in the first top clade of this phylogenetic tree.

Moreover, *in silico* MLST using the Pasteur database clustered all sequenced isolates as sequence types (STs) 243, while strain SVF3 has a novel ST assigned as * ff2b (Figure 3). The whole genome sequencing analysis revealed that all four isolates belong to *spa-type* t21. The four genomes were compared to African genomes reported from bovine milk (n = 38) and humans (n = 39). Comparative genomics showed that ST8500 and ST97 are prominent in milk bovine samples from Bloemfontein. Human isolates harbour ST-5 (*n* = 11), ST-152, and other variant sequence types. The four sequenced genomes in this study are 99.99% similar based on ANI analysis. Moreover, they cluster closely related to previously reported strain ma09 (ANI < 99.9%) isolated from bovine milk in Mofutsanyana, Free State. Furthermore, the cluster mentioned above groups with human isolates, i.e., strain DRKM31 and DRKM28, from a cutaneous abscess in Gauteng, South Africa (ANI < 96.8%) (Figure 4). Three clade groupings cater to most of the human *S. aureus*, while most bovine isolates form sub-clonal significant clades based on ANI profile.

### 3.5 Pangenomics analysis

**Figure 5:**
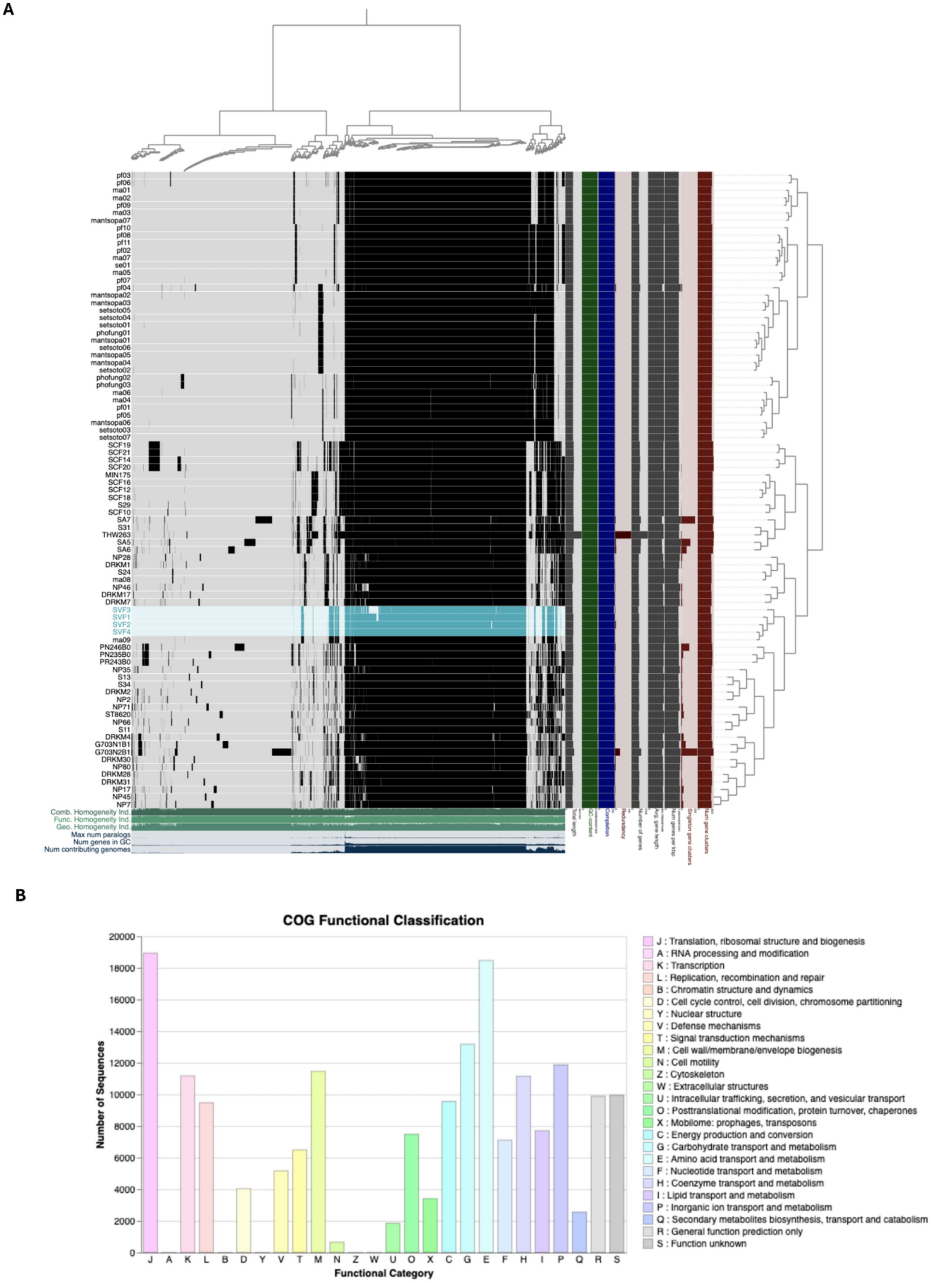
Pangenome visualisation of the *Staphylococcus aureus* (*n* = 85) genomes, including the four sequenced strains in this study. **(A)** Phylogeny of *S. aureus* indicates the core and accessory genes, placing the sequenced genomes highlighted in blue. Visualization of pangenome analyses carried out by Anvio. Central dendrogram clustering of samples is ordered by gene cluster presence/absence. Items order presence absence (D, Euclidean; L, Ward). **(B)** COG functional classification of the core genome of the analysed genomes.

A pangenomic analysis was performed on 85 *Staphylococcus aureus* genomes of South African origin, comprising four newly sequenced isolates from this study and 81 publicly available genomes (Figure 5). Using Roary, a total of 200,693 genes were identified across the dataset. Among these, approximately 1,144 genes were conserved across all genomes, constituting the *core genome*, which represents essential genetic elements shared among the strains. Phylogenetic reconstruction and gene presence/absence patterns indicate that the four newly sequenced isolates cluster closely with strain *ma09*, which was previously isolated from bovine milk in the Mofutsanyana district of the Free State Province. This close phylogenetic relationship strongly suggests localized dissemination of genetically similar *S. aureus* strains within the region, possibly driven by environmental or livestock-associated transmission dynamics once the toxin has been produced. Within this phylogenetic cluster, 2,293 core genes were identified, highlighting additional conserved genetic features unique to this regional lineage.

Notably, no upset gene clusters, defined as uniquely shared or absent gene combinations, were detected among the four sequenced isolates, indicating high genomic similarity and suggesting limited gene gain or loss events within this subgroup (Figure 5, Supplementary Figure S2). This observation supports the notion of a stable clonal lineage within the Free State Province. To complement Roary-based findings, an independent pangenomic analysis was performed using Anvi’o, which identified 5,098 gene clusters across the 86 genomes. This analysis reinforced the overall genomic consistency within the dataset and corroborated the absence of unique gene content differences among the four sequenced isolates. Functional annotation using the Clusters of Orthologous Groups (COG) database was applied to evaluate the distribution of gene functions across the dataset. Approximately 77.72% (181,522 out of 233,570 sequences) were successfully classified into COG functional categories (Figure 5B). The majority of the classified genes were involved in essential cellular processes, with the two most abundant categories being translation, ribosomal structure, and biogenesis (COG category J), comprising 10.4% of classified genes, and amino acid transport and metabolism (COG category E), comprising 10.1%.

### 3.6 Antibiotic resistance and virulence gene profiles

The antimicrobial resistance profile confidently identifies nine genes across four sequenced *S. aureus* strains (Supplementary Figure S1). All four isolates harboured the following seven genes: *LmrS, tet-38,* and *fosB* genes conferring resistance to lincomycin, a tetracycline efflux pump, fosfomycin resistance, and the more regulon, which is critical for virulence, biofilm formation, and antibiotic resistance. In addition, the presence of the *mec* operon regulatory components *arlS* and *arlR*, associated with oxacillin resistance and cell wall stress response, further underscores the multidrug-resistant potential of these strains. The detection of *mepA* and *mepR*, encoding a multidrug efflux pump and its transcriptional regulator respectively, highlights an additional mechanism of broad-spectrum resistance.

Importantly, the *norA* gene, which encodes a fluoroquinolone efflux pump, was uniquely identified in strains SVF 2 and SVF 4, suggesting a possible strain-specific adaptation to fluoroquinolone exposure. Moreover, the *more* regulon, which plays a pivotal role in virulence regulation, biofilm formation, and antibiotic resistance modulation, was detected in all four isolates, emphasizing their pathogenic potential (Peng et al., 2022). Whole genome sequencing profiling also confirms the absence of detectable plasmid incompatibility (Inc) types across all four genomes, suggesting that the resistance determinants are likely chromosomally encoded rather than plasmid-mediated. This finding may have implications for the persistence and spread of resistance genes in food-related *S. aureus* populations, suggesting a more stable integration of these traits.

In the case of the virulence genes, all isolates harboured the same 61 virulence-associated genes. (Supplementary Figure S2). These genes encompass a broad array of functions that collectively enhance the pathogen’s ability to colonize, evade host defenses, and cause disease. The distribution of these virulence determinants demonstrates notable variability among the isolates, suggesting strain-specific adaptations that may influence pathogenicity. Key adhesion-related genes were identified, including *clfA* (clumping factor A) and *spa* (protein A), which facilitate bacterial attachment to host tissues and immune components. Immune evasion mechanisms were supported by the presence of *sbi* (staphylococcal binder of immunoglobulin) and *spa*, both of which interfere with opsonization and phagocytosis. The genomes also harboured genes encoding cytolytic toxins such as *hla* (alpha-hemolysin) and *hlgA* (gamma-hemolysin component), which contribute to host cell lysis and tissue damage.

Biofilm formation capabilities were evident through the detection of the *icaADBC* operon, which is essential for polysaccharide intercellular adhesin (PIA) synthesis—an important feature for persistent infections and resistance to environmental stressors. Iron acquisition systems, including the *isdA* to *isdG* gene cluster, were also present in all strains, underscoring the importance of iron uptake during host colonization and infection. Furthermore, a suite of secreted proteases, including *sspA*, *sspB*, and *sspC*, was detected, supporting roles in tissue invasion, immune modulation, and nutrient acquisition. Notably, the SVF3 genome lacked two significant virulence determinants: *vWbp* (von Willebrand factor-binding protein), which contributes to immune evasion by interacting with host clotting factors, and *clfA*, an important fibrinogen-binding adhesin. The absence of these genes may reflect a reduced virulence capacity or niche-specific adaptation of this strain, which warrants further functional investigation.

## 4 Discussion

Foodborne pathogens pose a significant public health concern in South Africa, particularly in informal food markets where food safety regulations are often inadequate (Mphaga et al., 2024). Furthermore, this bacterium is becoming more concerning because of its resistance to antibiotics and further resulting in treatment and prevention difficulties (Brdová et al., 2024). In South Africa, street-vended foods are widely consumed due to their affordability and accessibility. Yet, they are often prepared under unhygienic conditions, creating an ideal environment for bacterial contamination and foodborne outbreaks.

Recently, several studies have been conducted on the prevalence of *S. aureus* isolates from raw meat and milk from South African Provinces such as Eastern Cape and Gauteng (Oguttu et al., 2014; Pekana and Green, 2018). Hence, a more comprehensive investigation of the prevalence of *S. aureus* isolated from ready-to-eat foods was needed. In this study, a total of 168 pap (maize meal), chicken, pork, and salad sold at the street food stalls in different municipalities of Mangaung Metro were subjected to an *S. aureus* investigation. Most of *S. aureus* isolates in the current study were detected from pork (36%) and chicken (32%). The results of this study are in agreement with those reported by Lin et al. (2009), who found that the predominant source of *S. aureus* isolates was pork samples, followed by chicken samples. *S. aureus* may infect chickens and pigs during breeding and slaughter, and as a result, the meat of these animals may become contaminated during sale, transportation, family storage, and cooking (Wang et al., 2017).

Out of 168 samples, 29.7% of samples were contaminated with *S. aureus*. This rate is lower than some similar studies done in Thailand, which recorded 78% samples contaminated with *S. aureus* from swab samples collected from the hands of food handlers (Tasanapak et al., 2023). A similar study in Northern Algeria recorded 21.4% (Achek et al., 2021), and 34.9% in Chinese retail meat (Wu et al., 2018). However, a lower prevalence of *S. aureus* was reported in studies conducted in southern Taiwan (Fang et al., 2003; Wei et al., 2006), and China (Yang et al., 2016). Other studies have reported the prevalence of *S. aureus* in ready-to-eat fruits and vegetables in the China (20.5%), Saudi Arabia (11.8%), Korea (1.8%) (Hasnah Hassan, 2011; Wang et al., 2019; Lin et al., 2019; Tango et al., 2018). *S. aureus* produces heat-stable toxins; therefore, food preparation processes such as cooking would not prevent staphylococcal poisoning once the toxin has been produced (Abdallah and Sulieman, 2024). The importance of educating consumers on food safety cannot be underestimated, as it is essential to reduce the incidence of foodborne illnesses and infections.

The study revealed high antimicrobial resistance among isolates, with 52% resistant to penicillin, 46% to cefoxitin, and 44% to ciprofloxacin, while lower resistance was observed for gentamicin (24%), erythromycin, and tetracycline (14%). However, they were less susceptible to gentamicin, erythromycin, and tetracycline, in that order. Although our study’s findings are not comparable to Wang et al. (2017) study, which demonstrated higher resistance to penicillin, tetracycline, and erythromycin, they are comparable to other research on raw food products in Spain (Rodríguez-Lázaro et al., 2017). Many antibiotics are also commonly used in veterinary medicine, particularly *β*-lactams (penicillin), macrolides (erythromycin), and lincosamide (clindamycin) (Cháfer-Pericás et al., 2010). In Bangladesh, 87 ready-to-eat meals yielded a total of 128 *S. aureus* isolates. Of these, 100 (78.1%) and 52 (40.6%) isolates were resistant to erythromycin and tetracycline, respectively (Xing et al., 2014), which is not comparable to our investigation.

Our findings further showed that the multidrug-resistant isolates were 21/50 (42%). These afore-mentioned percentages were not similar to those of a study conducted by Mahros et al. (2021), where they discovered that 75.2% of the isolates were categorized as MDR in Mansoura city. RTE foods may be a significant source of antibiotic resistance phenotypes, as evidenced by the high percentages of MDR staphylococci recovered from them. In the case of strains isolated from RTE food, the majority of isolates showed a resistance to cefoxitin which identifies methicillin-resistant strains that are phenotypically resistant to all beta-lactam antibiotics that have been used thus far in treatment, including carbapenems, penicillins, aminopenicillins, isoxazolyl penicillins (oxacillin, cloxacillin, dicloxacillin, and flucloxacillin), nafcillin, cephalosporins, penicillins, and cephalosporins with inhibitors (Gajic et al., 2022).

Out of the four isolates, whole genome sequencing identified three isolates as ST243, and one novel ST, and showed a close genetic relationship (99.99% ANI) with bovine and human *S. aureus* strains from South Africa, highlighting potential cross-host transmission dynamics. Notably, the four sequenced genomes are closely related to the ma09 strain isolated from bovine milk in Mofutsanyana, Free State and DRKM31. In contaminated agricultural settings, *S. aureus* isolates are primarily found in and transmitted to humans through farm animals, including cattle, pigs, and poultry (Kozytska et al., 2023). Meat and other animal products, such as dairy or eggs, may become contaminated during slaughtering and processing (Zhang et al., 2024). Consequently, individuals may unknowingly consume contaminated RTE products that contain *S. aureus*. Investigating *S. aureus* in a “One Health” context is crucial for understanding its genetic diversity, adaptive traits, and potential for antimicrobial resistance across human, animal, environmental and RTE sources.

As a rapidly growing bacterium, *S. aureus* requires an efficient protein synthesis machinery to thrive, particularly in nutrient-rich environments or host tissues (Bhattacharya and Horswill, 2024). The notable percentage of genes within COG J emphasizes the bacterium’s commitment to maintaining robust ribosomal and translational functions, which are vital for growth, adaptation, and survival during stressful conditions, including host immune responses (Shen et al., 2024). *S. aureus* relies significantly on effective nutrient acquisition systems, particularly for amino acids, to satisfy its metabolic needs, especially during infections when resources may be scarce (Richardson et al., 2019). These genes allow the bacterium to utilize amino acids for protein synthesis and as energy sources and precursors for various biosynthetic pathways (Halsey et al., 2017). The prominence of these functional classifications reflects *S. aureus*’s capacity to adapt to diverse environments, ensuring its survival and competitive edge in niches such as human hosts (Giulieri et al., 2022). These functions are essential for its pathogenesis, biofilm formation, and resistance mechanisms (Giulieri et al., 2022).

The ability of *S. aureus* to attach to host cells or extracellular matrix is a defining feature of its pathogenicity (Bhattacharya and Horswill, 2024). Adhesion is the initial stage of biofilm formation or host cell invasion, which shields bacteria from the immune system and promotes persistent infection (Pugazhendhi et al., 2022). Eight genes (*clfA, clfB, cna, fnbA, fnbB, srdC, srdD*, and *srdE*) encode a repertoire of surface proteins known as microbial surface components recognizing adhesive matrix molecules (MSCRAMMs), which are necessary for adhesion were detected in this study. The release of biofilm-related proteins depends on the expression of these genes (Foster, 2017). Bacterial pathogens’ capacity to form biofilms is thought to be antibiotic-resistant and linked to chronic infections in humans and animals (Singh et al., 2017). In *S. aureus* isolates sequenced in this study, nearly every gene in the *ica* operon was found. All sequenced *S. aureus* isolates included the *hlb*, *hld*, *hlgA*, *hlgB*, *hlgC*, and *hly* genes, which encode various hemolysins and leukocidins, which are virulence factors contributing to the bacterium’s pathogenicity by lysing red blood cells and immune cells (Zecconi and Scali, 2013). *Staphylococcus aureus* utilizes a sophisticated array of hemolysins and leukocidins to enhance its virulence by lysing host cells and evading immune responses. These factors are tightly regulated and play specific roles in different stages of infection, making them critical targets for potential therapeutic interventions. Understanding these mechanisms is essential for developing strategies to combat *S. aureus* infections (Zhang et al., 2018). Leukotoxin-encoding *luk* genes were present in all sequenced genomes in a study conducted by Budd et al. (2015) and Khasapane et al. (2024). The *luk* genes encode these leukotoxins, and their presence and expression vary among different *S. aureus* strains (von Eiff et al., 2004). However, human-derived isolates have also been reported to have *luk* genes (Enwuru et al., 2018).

Moreover, genomic analysis showed that the selected *S. aureus* isolates had been found to have MDR efflux pumps. Nonetheless, these components must be elevated to be connected to resistance (Papkou et al., 2020). It is noteworthy that the existence of genes that encode MDR efflux pumps, like *NorA*, has been linked to multiple virulence genes (De Gaetano et al., 2023). Specific virulence genes are more frequently found in *S. aureus* isolates with these MDR efflux pumps, while others are less common or completely absent in that subset of isolates. It is still being determined whether these patterns indicate a gene interaction that affects AMR and virulence in the absence of mechanical research. All known genetic factors related to antibiotic resistance and virulence factor genes and their interactions can be found throughout bacterial genomes. Furthermore, our results showed that the isolates had a *mepA* gene from the multi-antimicrobial extrusion (MATE) protein family, which is present in the *mepRAB* operon, possibly a multidrug transporter protein, according to a multidrug resistance phenotype seen in eight reported isolates (Costa et al., 2013).

This study also identified a tetracycline-resistant *tet*-38 gene that is a member of the Major Facilitator Superfamily (MFS) efflux pump genes. The chromosome-encoded *tet38* is a crucial membrane protein in *S. aureus* that helps explain the bacteria’s resistance to natural substances, including antibacterial fatty acids and antibacterial medications (Dashtbani-Roozbehani and Brown, 2021). The resistance of *S. aureus* to erythromycin and gentamicin is attributed to the *LmrS* efflux pump gene, which encodes the lincomycin resistance protein. The *LmrS* gene in this study revealed possible resistance to fenicol, oxazolone, and diaminopyrimidine (trimethoprim). Furthermore, Andersen et al. (2015) showed that the projected 47 kDa protein product, which consists of 14 transmembrane alpha-helices (TMH), was used by the *LmrS* gene activity to successfully extrude fusidic acid, kanamycin, lincomycin, and linezolid. The expression of the *norA* efflux pump gene, which regulates the ferric uptake regulator (*Fur*), and mutations occurring in the *norA* promoter area were linked to a chromosomally encoded resistance mechanism to ciprofloxacin (quinolone). Therefore, this study showed that 44% of all the *S. aureus* isolates investigated were indeed resistant to the antibiotics in question (Leroy et al., 2019). In this study, defensin (*mprF*), glycylcycline (*mepA*), daptomycin (*clsA*), fluoroquinolone and acridine dye (*arlS* and *arlR*), glycylcycline (*mepA*), and diaminopyrimidine (*dfrC*) were among the genes that were found in the *S. aureus* genomes. These genes aligned with earlier research conducted by Naorem et al. (2021), which also discovered genes linked to antibiotic resistance in *S. aureus* isolated from the food industry, bovine milk and clinical samples (Ajose et al., 2024).

This study offers valuable insights into the prevalence, antimicrobial resistance, and virulence potential of *S. aureus* isolated from ready-to-eat foods in the Mangaung Metro. However, a notable limitation is that not all isolates underwent whole genome sequencing; only four representative isolates were sequenced. This may not adequately reflect the genomic diversity, resistance determinants, or virulence gene profiles present across the isolates investigated. Consequently, the findings regarding sequence types, antimicrobial resistance genes, and virulence factors may not be applicable to all *S. aureus* strains found in these food sources. Moreover, another limitation is the relatively small number of isolates (*n* = 50) utilized for antimicrobial resistance analysis. The limited sample size was due in part to the loss of some isolates during storage and financial constraints that restricted the number of tests conducted. Other limitations of this study are the lack of PCR-based screening for staphylococcal enterotoxins in the food samples. Although the whole genome sequencing of the four representative isolates did not identify any enterotoxin-encoding genes, the absence of direct enterotoxin detection prevents us from drawing definitive conclusions about their presence or absence in the broader set of isolates analyzed in this study. Future research that expands the whole genome sequencing to include a larger number of isolates would improve our understanding of the molecular epidemiology and potential public health risks related to foodborne *S. aureus*.

## 5 Conclusion

This study has identified a significant presence of antimicrobial resistance profiles of *S. aureus* from ready-to-eat foods in Mangaung Metropolitan Municipality. Of concern is the issue of having over 50% of the isolates being MDR. Furthermore, the study has also managed to detect the presence of virulence genes, namely leucocidin, hemolysin, and aureolysin, along with antimicrobial resistance (AMR) genes such *as lmrS, mepA*, and *tet (38)*. These results suggest that contaminated food may play a role in disseminating drug-resistant strains of *S. aureus*. To combat this issue, it is crucial to establish a targeted education and awareness campaign alongside a comprehensive surveillance program for *S. aureus* throughout the entire food production and supply chain, with a particular emphasis on industries that produce RTE foods. Additionally, the findings from this study may hold broader implications for developing nations and other regions where RTE food is sold and consumed. Gaining a deeper understanding of the epidemiology of *S. aureus* genotypes in ready-to-eat (RTE) food is essential for developing effective treatment and control strategies to curb the spread of this pathogen.

## Data Availability

The datasets presented in this study can be found in online repositories. The names of the repository/repositories and accession number(s) can be found in the article/Supplementary material.

## Footnotes

1
https://pathogen.watch/

2
https://www.genomicepidemiology.org/

3
https://www.bv-brc.org/
