# Supplementary material for: Antimicrobial resistance and genomic characterization of Staphylococcus aureus in ready-to-eat foods from Mangaung Metro Municipality
Source: Front Microbiol. 2025 Dec 5;16:1669035. doi: 10.3389/fmicb.2025.1669035 (PMC12714933; doi:10.3389/fmicb.2025.1669035)
Supplement: Supplementary file 2 [file Table_2.DOCX]

Antimicrobial resistance and genomic characterization of *Staphylococcus aureus* in ready-to-eat foods from Mangaung Metro Municipality

Pontso Letuka^1^, Sebolelo J Nkhebenyane^1^, Tsepo Ramatla^1^, Tywabi-Ngeva Zikhona^2^, Kgaugelo E Lekota^3^, Ntelekwane G Khasapane^1^*

^1^Department of Life Sciences, Centre for Applied Food Safety and Biotechnology, Central University of Technology, Bloemfontein, South Africa.

^2^Department of Chemistry, Nelson Mandela University, Port Elizabeth 6001, South Africa.

^3^Unit for Environmental Sciences and Management, North-West University, Potchefstroom, South Africa.

*** Correspondence:**Ntelekwane G Khasapane
[nkhasapane@cut.ac.za](mailto:nkhasapane@cut.ac.za)

**Supplementary Table S1.** MAR index of *Staphylococcus* spp. Isolates

| **Isolate No** | **No. of antibiotics to which isolate was resistant (*a*)** | **MAR index = *a*/*b*** |
| --- | --- | --- |
| P1 | 5 | 0.83 |
| C15 | 4 | 0.66 |
| PK37 | 5 | 0.83 |
| S47 | 5 | 0.83 |


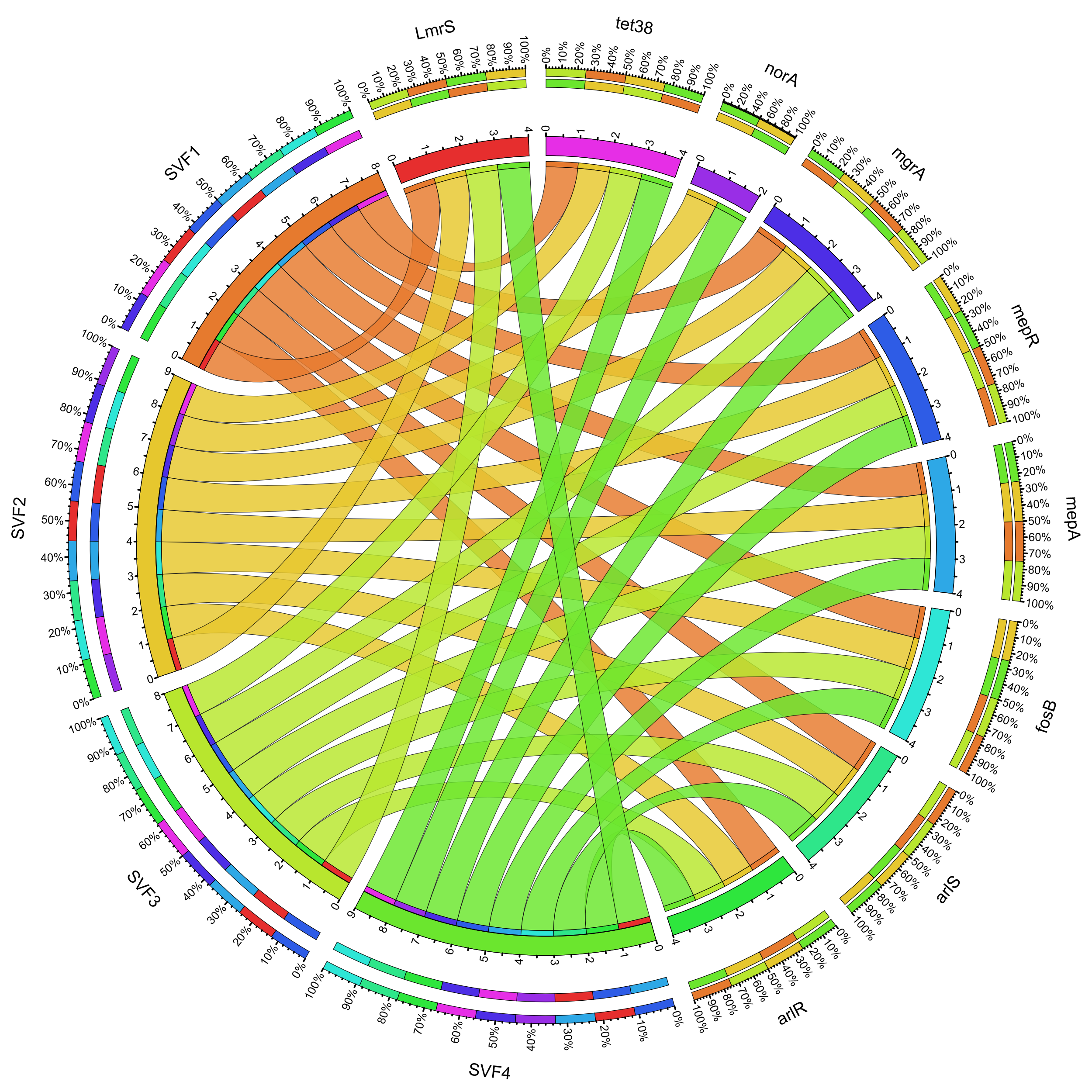


**Supplementary figure S1.** Antibiotic resistance gene profiles of the four sequenced *S. aureus* isolates from food determined using the CARD database. Each colour code lines of strain number i.e. SVF1, SVF2, SVF3, and SVF4 correspond to the gene and presence of each ARGs.


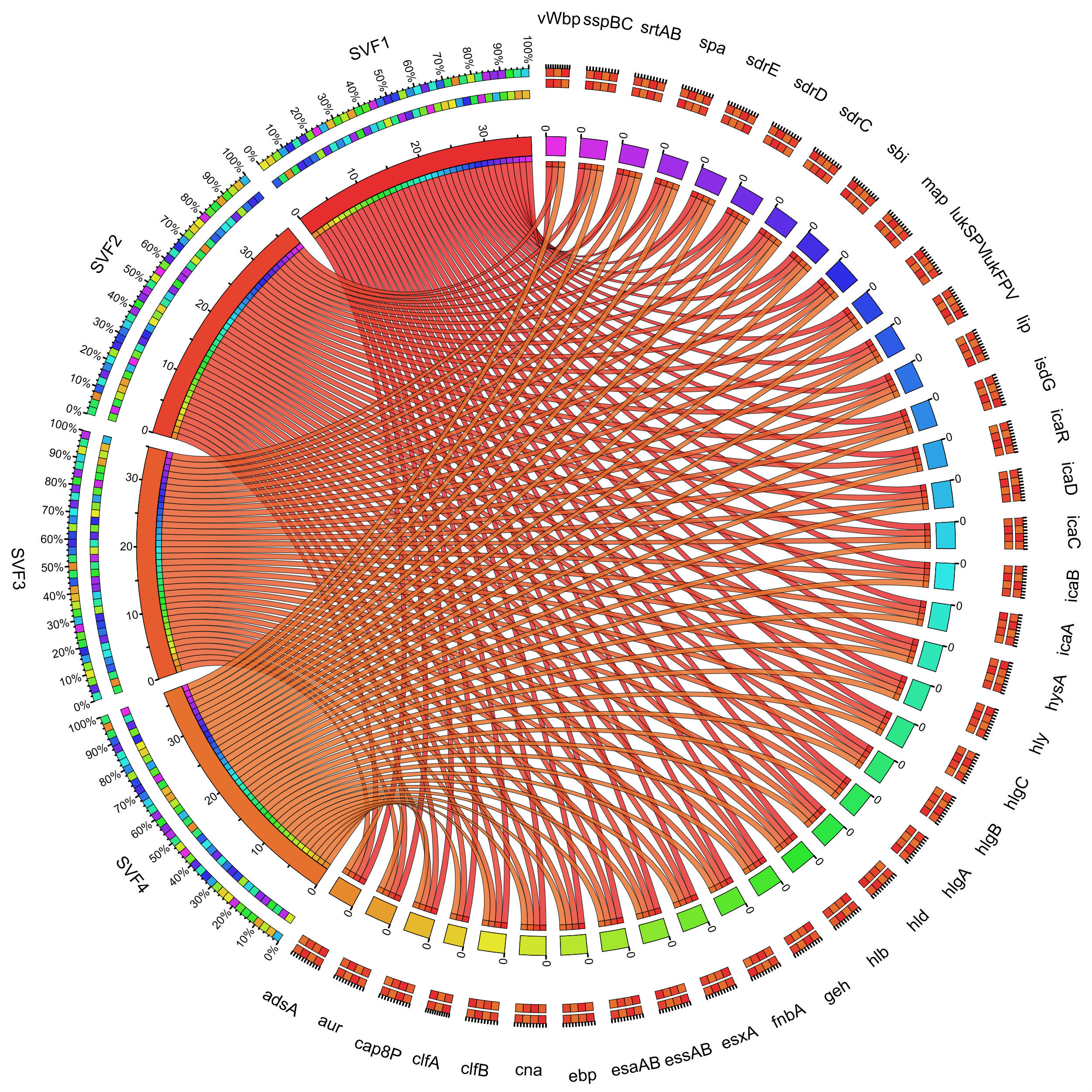


**Supplementary figure S2:** Virulence gene profiles of the four sequenced *Staphylococcus aureus* isolates from food determined using the virulence factor database. Each colour code lines of strain number i.e. SVF1, SVF2, SVF3, and SVF4 correspond to the gene and presence of each virulent genes.
